# IMPACT OF DIGITAL AND SOCIAL ENGAGEMENT ON QUALITY OF LIFE AMONG RURAL OLDER ADULTS

**DOI:** 10.1093/geroni/igad104.2727

**Published:** 2023-12-21

**Authors:** Jeehoon Kim, Gesine Hearn, Anna Hall

**Affiliations:** Idaho State University, Pocatello, Idaho, United States; Idaho State University, Pocatello, Idaho, United States; Idaho National Laboratory, Idaho Falls, Idaho, United States

## Abstract

**Objectives:**

In the current digitalized society, digital and social engagement are important elements in the well-being of older adults. This study examines how different types of digital and social engagement are associated with different aspects of quality of life among rural healthy older adults.

**Methods:**

Older adults without cognitive impairments, residing in the community in Southeast Idaho (n=154) participated in the ‘Aging in Idaho study’ in 2018. Internet use for communication, instrumental purposes, and health information searches were measured. Informal and formal social participation were measured. Five aspects of quality of life (QOL) were measured using the WHOQOL-BREF. Multiple regression analyses were performed.

**Results:**

Well-educated (M=16 years) rural older adults used the Internet for various purposes, actively engaged in social activities, and reported higher level of QOL for overall and life environment (e.g., M=4.6, Range=1-5). Internet use for communication was positively associated with QOL general health (β=.20, P <.05). Online health information search was negatively associated with QOL general health (β= -.42, P <.05). Informal social participation was positively associated with QOL overall (β=.17, P <.05), psychological health (β=.13, P <.05), and life environment (β=.20, P <.05). Formal social participation was positively associated with QOL social relationships (β=.16, P <.05).

**Discussion:**

Digital and social engagement relate in distinct ways with different aspects of QOL. Older adults perceived QOL to be better when they actively adopted digital technology whereas maintaining social engagement well in the community. Promoting digital engagement which enables to facilitate social engagement in later life will be discussed.

